# Supplementary material for: Combining a prioritization strategy and functional studies nominates 5’UTR variants underlying inherited retinal disease
Source: Genome Med. 2024 Jan 6;16:7. doi: 10.1186/s13073-023-01277-1 (PMC10771650; doi:10.1186/s13073-023-01277-1)
Supplement: Supplementary file 2 — Additional file 2: Figure S1. Schematic overview of constructs used for functional studies. Figure S2. Assessment of transcription start site (TSS) confidence by CAGE-seq in retina. Figure S3. Retina-enriched non-canonical isoforms of the CRB1 and RIMS2 genes. Figure S4. Evaluation of 5’UTR capture by the kits mostly used for generating our in-house WES data. Figure S5. Pedigrees of families carrying the 11 candidate 5’UTR variants and segregation analysis. Figure S6. Secondary structure prediction for wild-type and mutated 5’UTR sequences. Figure S7. Kozak consensus sequence of all IRD gene isoforms evaluated in this study. Figure S8. Sample overview of qPCR-based analysis of ARL3 levels in ARL3:c-88G>A heterozygotes. Figure S9. Characterization of the 5’UTR splice variant c.-9+1G>T in PRPF31. [file 13073_2023_1277_MOESM2_ESM.pdf]

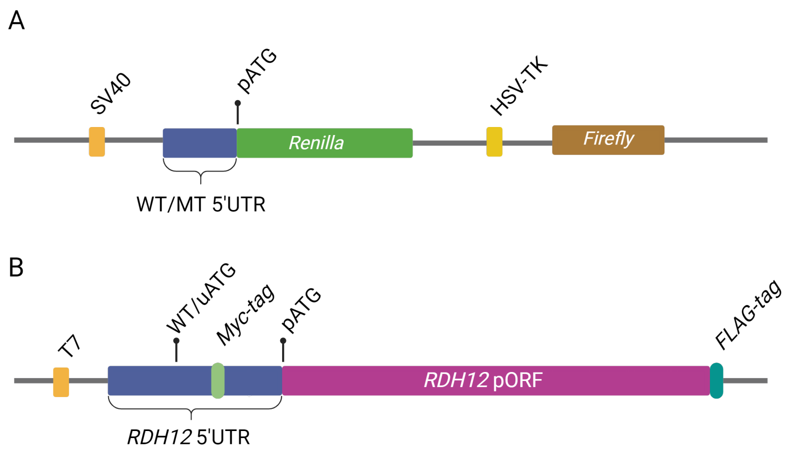

Figure S1. Schematic overview of constructs used for functional studies. (**A**) The wild-type (WT) 5’UTRs of the *MERTK*, *PAX6*, and *RDH12* genes were cloned in-frame into a psiCHECK™-2 dual luciferase vector (Promega), containing the *Renilla* and *Firefly* luciferase reporter genes under the regulation of the SV40 and HSV-TK promoters, respectively. (**B**). Depiction of the overexpression RDH12 construct comprising the 5’UTR and primary open reading frame (pORF) of *RDH12*, for which Myc and FLAG in-frame tags were included downstream, respectively. This fragment was cloned into a pcDNA™3.1$^{(+)}$ (Invitrogen) vector downstream its T7 promoter. For all constructs, 5’UTRs variants were created by site-directed mutagenesis to obtain mutant (MT) constructs with the variants of interest.

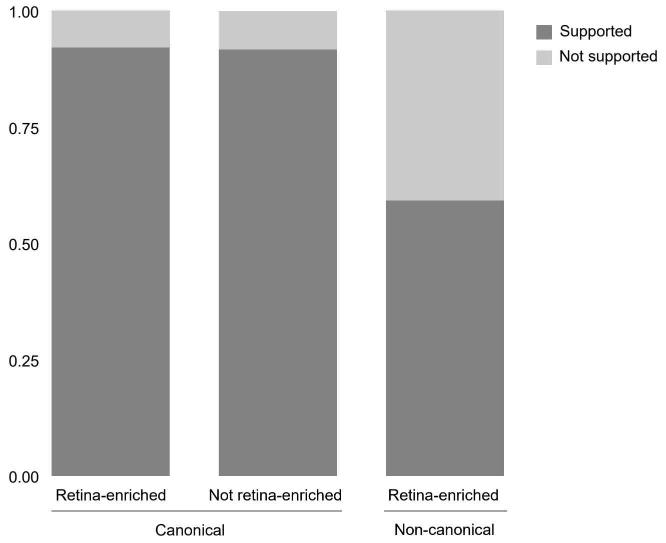

Figure S2. Assessment of transcription start site (TSS) confidence by CAGE-seq in retina. Representation of the proportion of IRD gene isoforms based on the support of their annotated TSS provided by CAGE-seq data derived from adult and fetal retina.

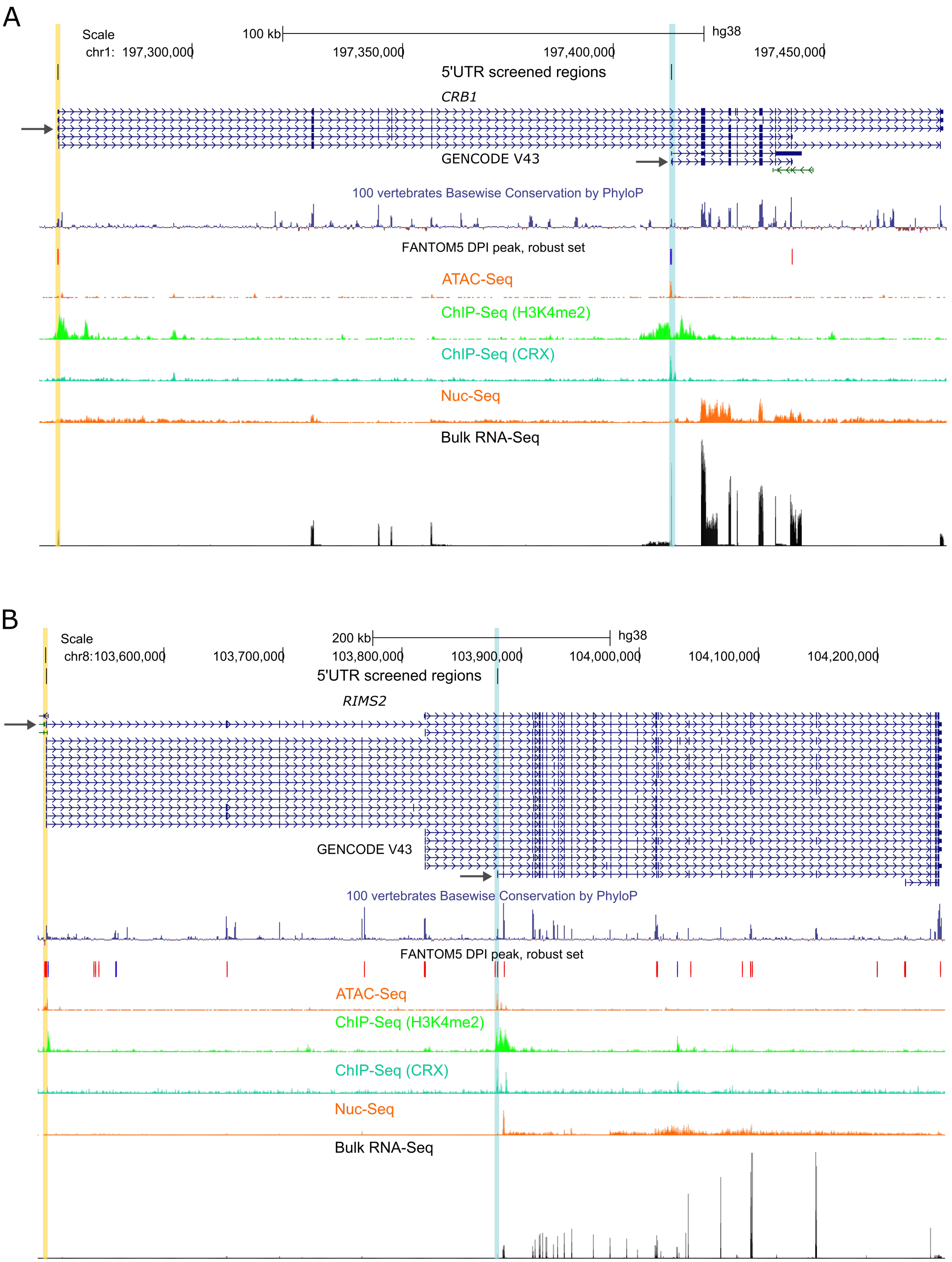

Figure S3. Retina-enriched non-canonical isoforms of the *CRB1* and *RIMS2* genes. The shorter alternative isoforms of (**A**) *CRB1* (ENST00000681519) and (**B**) *RIMS2* (ENST00000436393) contain 5'UTRs which are completely distinct to those of their respective canonical isoforms (ENST00000367400.8 and ENST00000696799.1, respectively). The transcription start sites (supported by CAGE-seq) of each canonical and non-canonical isoform (indicated by arrows) are highlighted in *yellow* and *blue*, respectively. Active retinal transcription is supported by bulk RNA-seq and Nuc-seq derived from human retina. The enrichment in retina of these isoforms is further supported by signatures of open chromatin, H3K4me2, and binding of the retina-specific transcription factor CRX (datasets used are listed in **Table S2**).

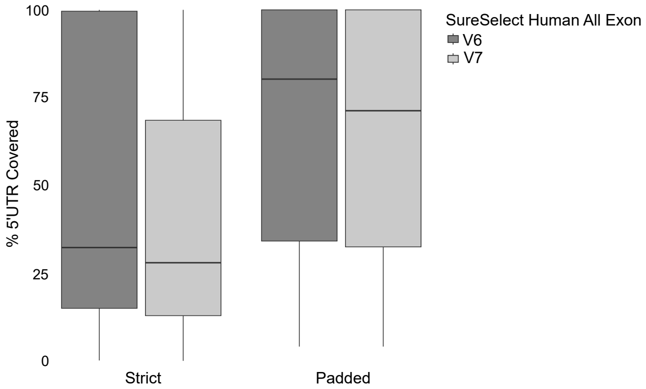

Figure S4. **Evaluation of 5'UTR capture by the kits mostly used for generating our in-house WES data**.

Comparison of the capture performance of the SureSelect Human All Exon V6 and V7 kits (Agilent Technologies) considering *strict* or *padded* designs (*see Methods*).

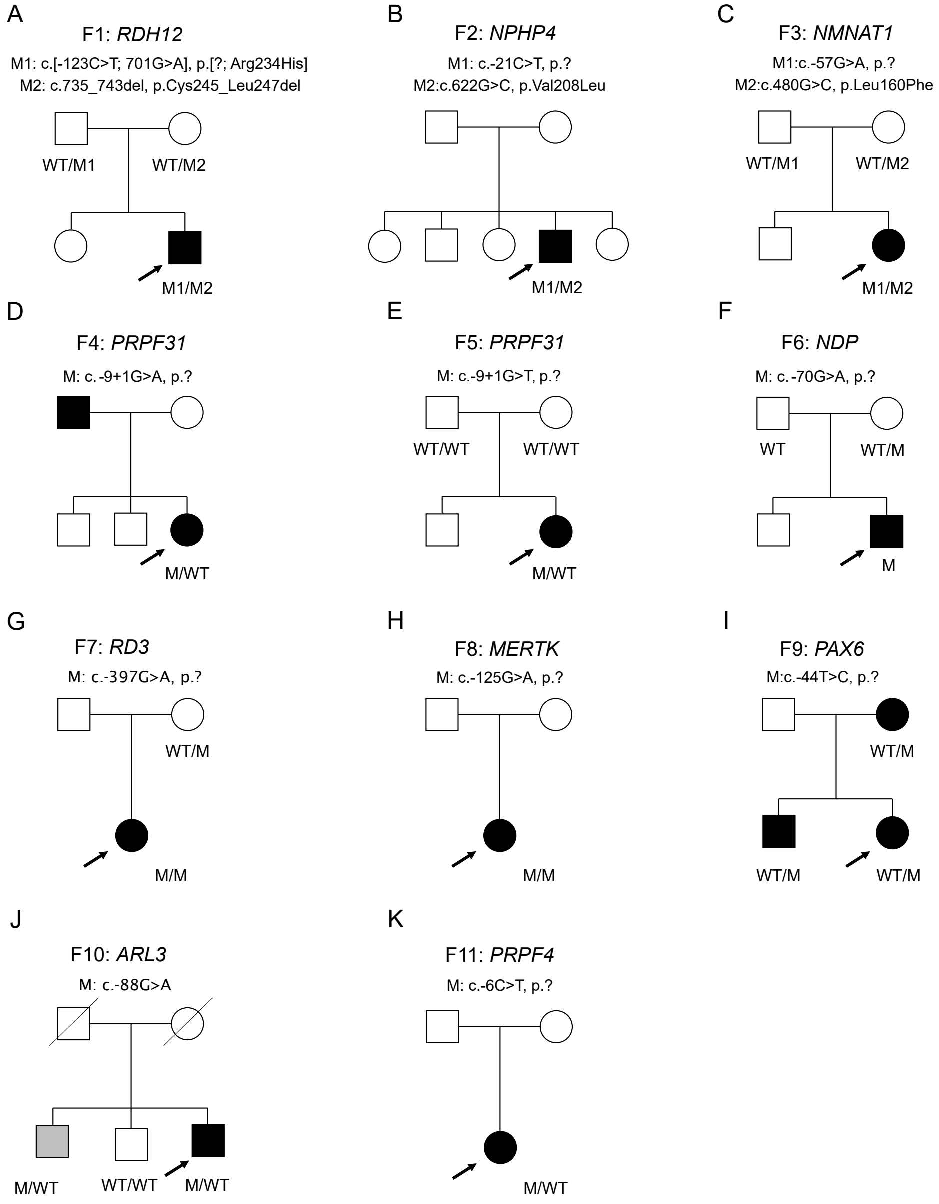

Figure S5. Pedigrees of families carrying the 11 candidate 5'UTR variants and segregation analysis.

(**J**) Upon clinical re-evaluation, the sibling of F10 -*gray*- was found to be affected by an acquired vascular ocular condition instead of an IRD. Abbreviations: WT, wild-type allele; M, mutated allele.

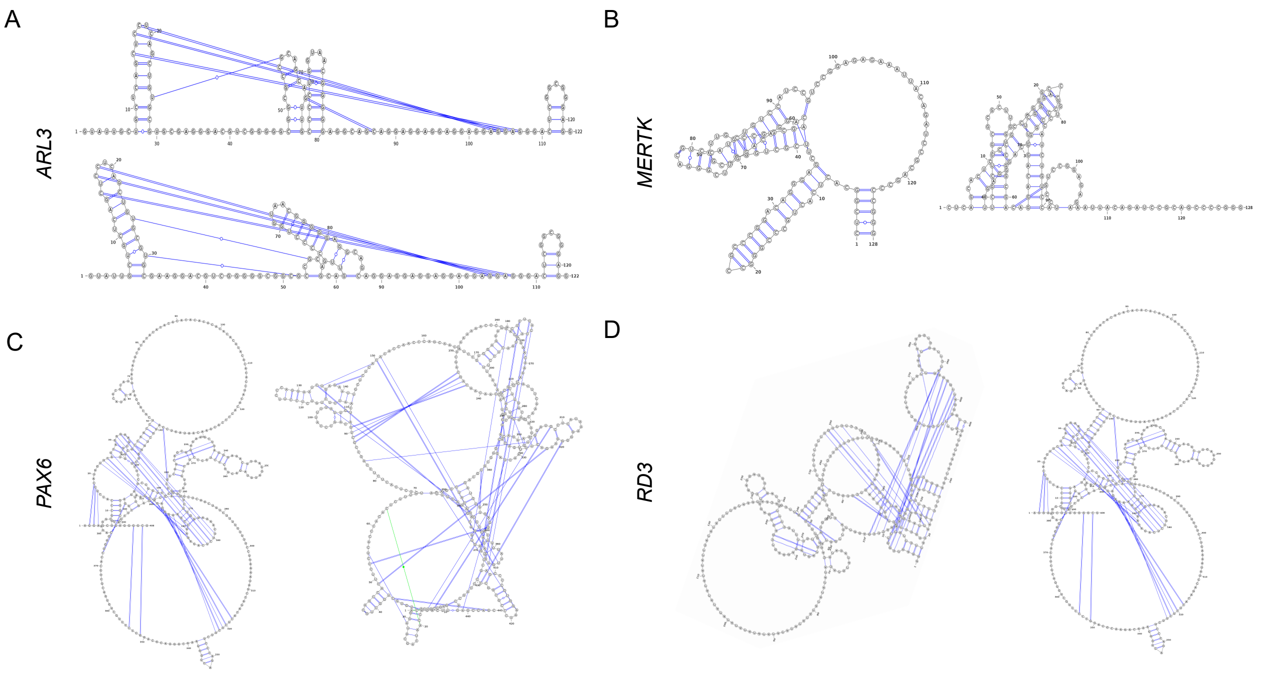

Figure S6. Secondary structure prediction for wild-type and mutated 5'UTR sequences. Secondary structure analysis of the 5'UTR sequences (*Ufold* using default parameters) revealed different folding for the **(A)** *ARL3*:c.-88G>A, **(B)** *MERTK*:c.-125G>A, **(C)** *PAX6*:c.-44T>C, **(D)** *RD3*:c.-394G>A variants in comparison to their corresponding wild-type 5'UTRs.

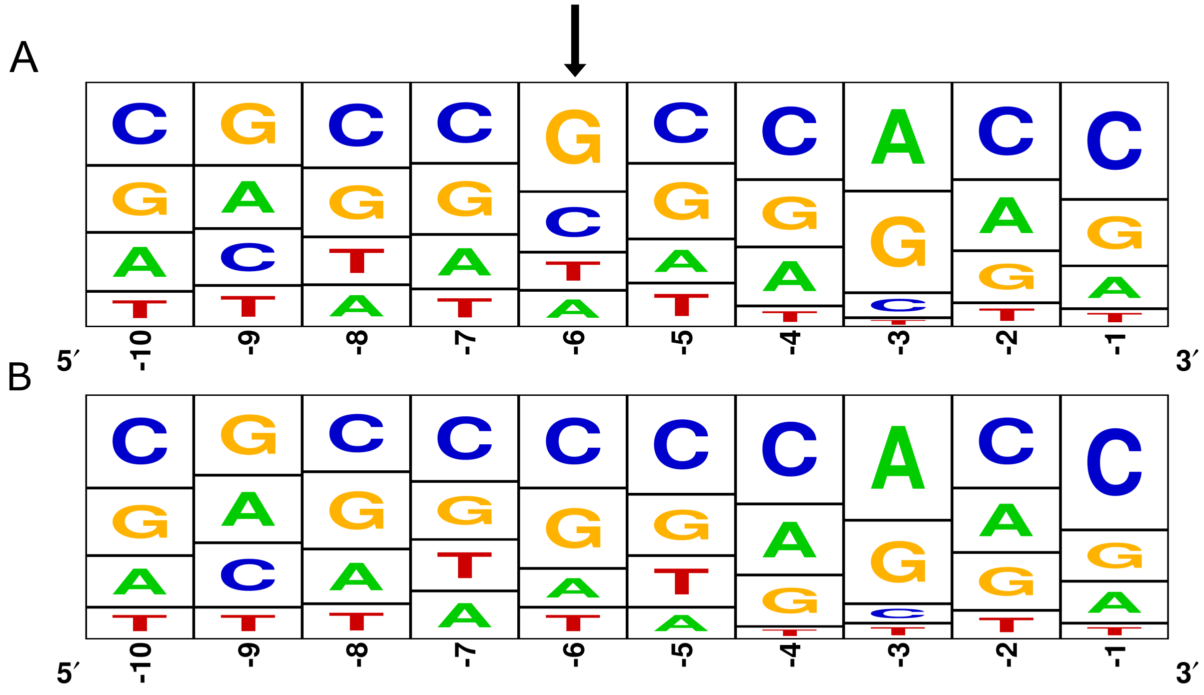

Figure S7. Kozak consensus sequence of all IRD gene isoforms evaluated in this study. The nucleotides within the -1 to -10 positions relative to the main AUG were retrieved for all selected transcripts. Frequency plots corresponding to the Kozak sequences of the (**A**) retina-enriched (373) and (**B**) not retina-enriched canonical transcripts (76).

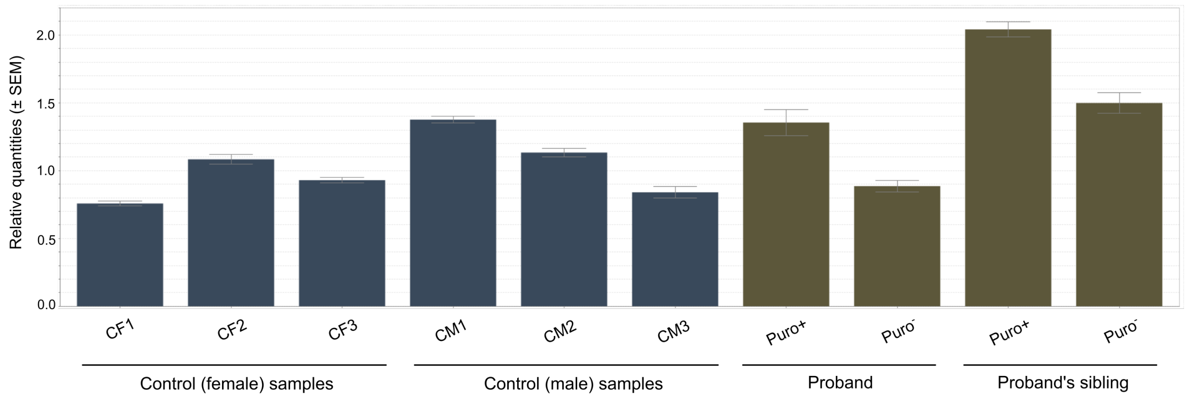

Figure S8. Sample overview of qPCR-based analysis of *ARL3* levels in *ARL3*:c-88G>A heterozygotes. qPCR-based quantification of relative *ARL3* mRNA abundance in lymphocyte cDNA of *ARL3*:c-88G>A carriers (proband and sibling) and 6 healthy controls (3 females and 3 males). Data are scaled to the average across all control samples. Error bars represent the standard error of three qPCR technical replicates.

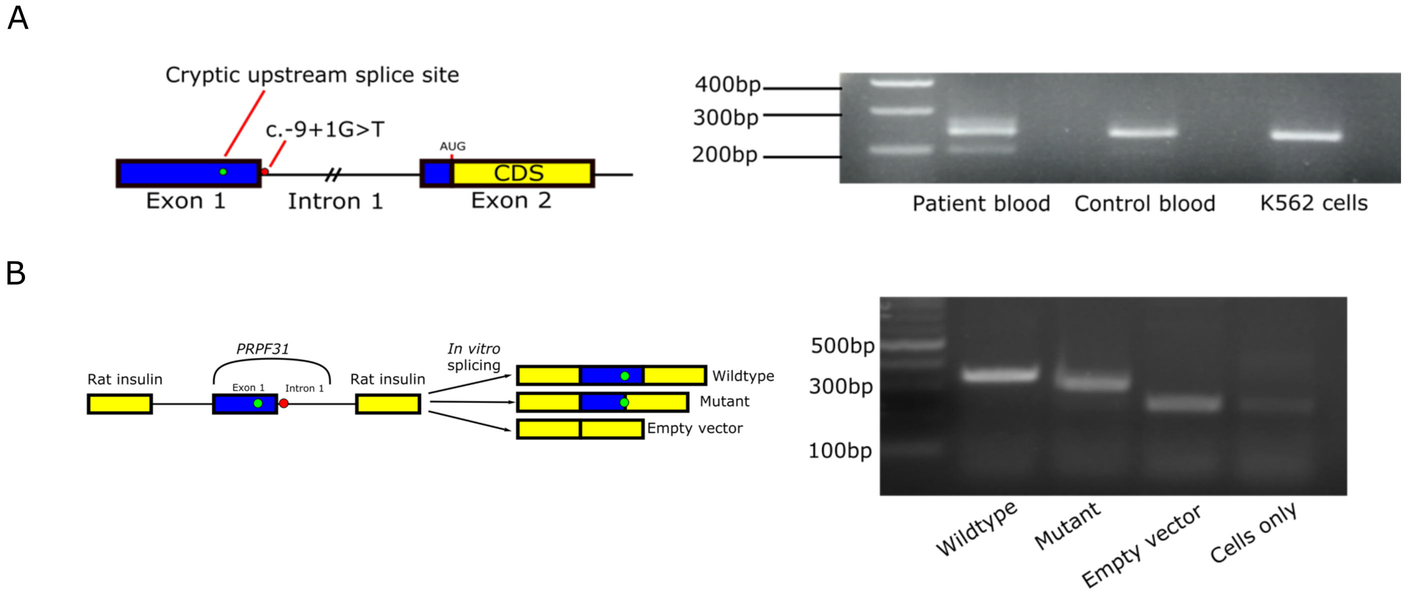

Figure S9. Characterization of the 5'UTR splice variant c.-9+1G>T in *PRPF31*. (**A**) Schematic depiction of the 5' end of the *PRPF31* gene (*left*) and representative RT-PCR showing the impact of the variant on splicing of *PRPF31* in RNA isolated from patient blood compared to control blood and human K562 cell lines (*right*). The shorter amplicon present in the patient sample corresponds with recognition of an upstream cryptic donor splice site in exon 1. (**B**) Schematic outlining the *PRPF31* minigene construct (*left*) and outcomes of splicing *in vitro* (*right*), confirming a shorter fragment in the mutant vector. Abbreviations: CDS, coding sequence.
